# Evaluation of the Awareness of Antidepressant Drug Usage Among the Population in Madinah: A Cross-Sectional Study

**DOI:** 10.7759/cureus.101218

**Published:** 2026-01-10

**Authors:** Faisal A Almutairi, Abdullah F Almohammadi, Abdulaziz N Alsehli, Asim M Alsahli, Hazim I Alraddadi, Omar A Alzahrani, Waleed H Alharbi, Yazeed A Habeeb, Ahmed S Metwally

**Affiliations:** 1 Family Medicine, Community Medicine, and Medical Education, College of Medicine, Taibah University, Madinah, SAU; 2 Family Medicine, Suez Canal University, Ismailia, EGY

**Keywords:** antidepressants, awareness, depression, madinah, saudi arabia, stigma

## Abstract

Introduction: Depression is common, and antidepressants are widely used, yet a poor understanding of antidepressant medications may contribute to hesitancy in seeking help and non-compliance with treatment. Public understanding and attitudes toward these drugs, especially in Madinah, remain unclear.

Objectives: This study aimed to assess the level of awareness and identify demographic and exposure-related factors influencing public perceptions of antidepressant use among adults residing in the Al-Madinah Region, Saudi Arabia.

Methods: This was an observational, analytical cross-sectional study conducted using an online self-administered questionnaire among adults (≥18 years) residing in the Al-Madinah Region, Saudi Arabia. A non-probability convenience sampling approach was used, and a total of 427 participants were included. The questionnaire was adapted from a previously published instrument and pilot-tested to ensure clarity and flow prior to full data collection. The questionnaire included demographic and exposure items and 12 statements assessing knowledge and beliefs about antidepressant use, scored 0-12 (higher scores indicating more evidence-consistent and less stigmatizing responses). Data were analyzed using chi-square/Fisher’s exact tests and t-tests/ANOVA, as appropriate; p≤0.05 was considered statistically significant.

Results: The study showed several misconceptions about antidepressant use: 73.5% believed that depression could resolve on its own, and 65.7% believed that antidepressants work immediately. Concerns about addiction (74.5%) and long-term use (45.7%) were also common. Higher awareness was significantly associated with being female (45.5%) and having a university education (43.1%) (p < 0.05). Regarding attitudes, 67.9% disagreed that antidepressant use indicates personal weakness, while 30.4% believed that antidepressant use could negatively affect employment opportunities.

Conclusion: These findings highlight a clear gap in public understanding and the need for awareness efforts through education, media, and community initiatives to address misconceptions and reduce stigma.

## Introduction

Depression is a common mood disorder characterized by persistent low mood and loss of interest. The 12-month prevalence of major depressive disorder is approximately 7% worldwide [1], and it has been reported to be about 17% in Saudi Arabia [2]. Given its burden on individuals and communities, improving understanding of depression and its treatments remains an important public health priority.

Major depressive disorder has a complex etiology involving biological, genetic, environmental, and psychological factors. One proposed biological mechanism is dysregulation of key neurotransmitters-particularly serotonin, norepinephrine, and dopamine-which is relevant to the mechanisms of many antidepressant medications, although it does not fully explain the multifactorial nature of depression [3]. This background helps explain why antidepressants are widely used as part of treatment.

Depression management includes psychological and pharmacological approaches. Treatment often begins with psychological therapy, and antidepressant medication may be added or used when clinically indicated [4]. As a result, antidepressants are frequently encountered in clinical practice and in the general community.

Antidepressants are used not only for depressive disorders but also for other psychiatric conditions such as generalized anxiety disorder, post-traumatic stress disorder, phobias, panic disorder, and obsessive-compulsive disorder [5]. Common classes include selective serotonin reuptake inhibitors (SSRIs), serotonin-norepinephrine reuptake inhibitors (SNRIs), heterocyclic antidepressants, monoamine oxidase inhibitors (MAOIs), and other atypical agents [5,6]. Their broad indications and increasing use make public knowledge and attitudes toward these medications particularly relevant.

While antidepressants can be effective, they may cause adverse effects that influence acceptance and adherence. For example, SSRIs can be associated with sexual dysfunction, drowsiness, weight changes, sleep disturbances, dizziness, and headaches [7], and sexual adverse effects are commonly reported in both men and women [8]. Concerns about side effects, dependence, and stigma may contribute to hesitancy in seeking care, premature discontinuation, or misconceptions about treatment.

Public understanding of antidepressants can influence help-seeking behavior, treatment adherence, and stigma, yet awareness and attitudes may vary by setting and may be influenced by education and psychosocial barriers. A study from Riyadh reported gaps in antidepressant awareness and misconceptions, with education identified as a significant factor associated with awareness [9]. Other studies have highlighted psychological barriers to treatment-seeking even among individuals who recognize a need for care [10], and an association between lower educational level and lower mental health awareness in some contexts [11]. However, local data on antidepressant awareness and attitudes among adults in the Al-Madinah region are limited. Therefore, this study assessed public knowledge, common misconceptions, and attitudes toward antidepressant use among adults residing in Al-Madinah, Saudi Arabia.

Aim and objectives

The aim of this study was to evaluate public knowledge and attitudes toward antidepressant use among adults residing in the Al-Madinah Region, Saudi Arabia. The objectives of this study were to assess the knowledge and common misconceptions about antidepressant use, to assess the attitudes toward antidepressant medications, and to examine how these outcomes vary according to participant characteristics (e.g., sex, age group, education level, medical background, personal/family history of depression or antidepressant use, and prior exposure to information about antidepressants). Our study hypotheses: Null hypothesis (H0) stated that the participants with higher education or a family history of depression and/or antidepressant use do not have greater knowledge and more positive attitudes toward antidepressant use compared with participants without these characteristics. Alternative hypothesis (H1) stated that the participants with higher education or a family history of depression and/or antidepressant use have greater knowledge and more positive attitudes toward antidepressant use compared with those without these characteristics.

## Materials and methods

Study design and settings

This was an observational, analytical cross‑sectional study conducted using an online self‑administered questionnaire (see Appendix). The study targeted adults (≥18 years) residing in the Al‑Madinah Region, Saudi Arabia. Data collection occurred during the period 05/09/2024-05/11/2024. 

Participants and eligibility criteria

Participants were selected according to predefined criteria. Inclusion criteria were individuals from the general population aged 18 years or older and residing in the Al-Madinah region of Saudi Arabia. Exclusion criteria included individuals younger than 18 years, those residing outside the Al-Madinah region, and individuals who declined participation to be included in the study after explanation of the study.

Sampling strategy and recruitment

A non-probability convenience sampling approach was used. The survey link was disseminated online through community networks and digital platforms accessible to the general public. Eligibility screening questions were presented at the beginning of the survey, and only eligible respondents could proceed. Because recruitment relied on an open online link and the number of individuals who received or viewed the invitation is unknown, a response rate could not be calculated. As the sampling was non-probabilistic, findings should be interpreted as descriptive of the surveyed respondents and not statistically representative of the entire Al-Madinah population.

Sample size

In total, 427 participants were recruited for this study. The required sample size was estimated using the standard sample size formula for proportions: n = P(1 − P) × Z² / d². For this calculation, Z was set at 1.96, corresponding to a 95% confidence level, P was assumed to be 0.50 to represent maximum variability, and the allowable margin of error (d) was set at 0.05. Substituting these values resulted in n = (1.96)² × 0.50 × 0.50 / (0.05)² = 384 participants [12]. To account for incomplete or unusable responses, recruitment continued beyond the minimum; 427 complete responses were included in the final analysis.

Study instrument and scoring

Data were collected using a questionnaire adapted from a previously published study conducted in Riyadh [9]. The questionnaire involves 20 questions. The questionnaire included three sections: Demographic factors, knowledge about depression, The attitude toward antidepressant medications. Knowledge about antidepressants was assessed using "12" questions with responses categorized as either "agree, maybe, don't agree, and don’t know." Each correct answer was assigned a score of "1", while incorrect answers were scored as "0". For descriptive comparison with prior work, we also created a secondary dichotomous classification: scores 0-6 (lower awareness) and 7-12 (higher awareness). This cutoff represents more than half of the items answered in the evidence‑consistent direction and is treated as a descriptive/secondary analysis.

Questionnaire language and pilot testing

The questionnaire was administered in Arabic and English. The Arabic version was prepared by bilingual investigators and reviewed for clarity. A pilot test (n = 40) was conducted to confirm comprehensibility and flow; minor adjustments were made to question order for readability. Formal psychometric validation beyond adaptation from prior work was not performed.

Statistical analysis

The collected data were entered and analyzed using the IBM SPSS Statistics for Windows, version 22.0 (released 2013, IBM Corp., Armonk, NY) [13]. Categorical data were presented as frequencies. The distribution of knowledge items among the participants was described using frequencies and percentages. Knowledge levels (good vs. poor) were compared based on the characteristics of the participants using chi-square tests and Fisher’s exact test, as appropriate. Chi-square assumptions checked; Fisher’s exact used when expected counts <5. In addition, the mean knowledge scores were compared based on the participants' characteristics using independent t-tests and analysis of variance (ANOVA). A p-value of < 0.05 was considered statistically significant. Degrees of freedom (df) were reported for t-tests and ANOVA (F(df1, df2) for ANOVA; t(df) for two-group comparisons).

Ethical considerations

Ethical approval was obtained from the Scientific Research Ethics Committee at Taibah University (approval no. IRB00010413). Participants provided electronic informed consent prior to participation. Responses were anonymous and collected without personal identifiers. Participation was voluntary and participants could exit the survey at any time.

## Results

**Figure 1:**
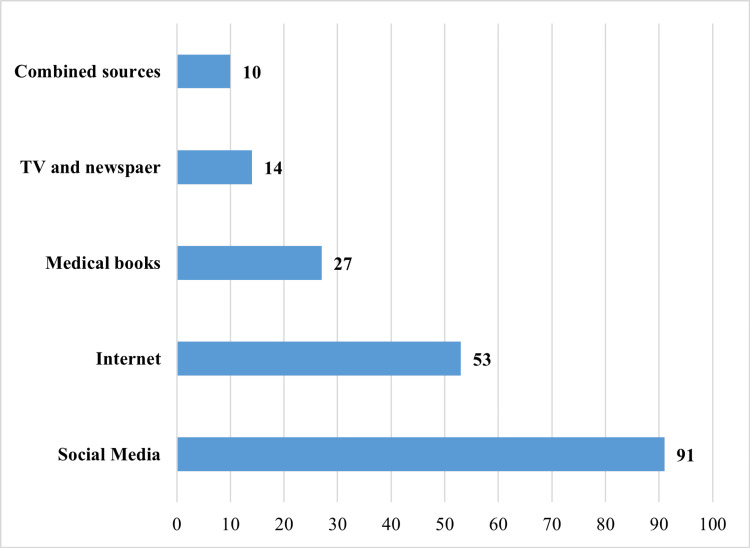
Sources of information about antidepressants among participants who previously learned or read about antidepressants This figure shows where participants who reported prior learning/reading about antidepressants obtained their information. This was a multiple-response item (select all that apply). Participants selecting more than one source are grouped under “Combined sources.” Values are presented as frequencies.

Table 1 summarizes the participant characteristics (N = 427). Participants were mainly aged ≥45 years (39.5%) or 18-25 years (31.9%), with a slight female majority (54.1%) and predominantly university education or higher (78.2%). A minority reported a medical/healthcare background (17.6%) or prior depression diagnosis/treatment (13.6%); 33.7% reported a relative treated with antidepressants, and 45.7% (n = 195) reported having previously read/learned about antidepressants, with information sources shown in Figure 1. 

**Table 1:** Characteristics of the studied participants Participant characteristics (N = 427), including age group, sex, educational level, medical background, depression history, family exposure to antidepressants, and prior learning/reading about antidepressants; values are presented as n (%).

Characteristics	N = 427
Age in years	
18-25	136 (31.9%)
26-35	43 (10.1%)
36-45	79 (18.5%)
45+	169 (39.5%)
Sex	
Male	196 (45.9%)
Female	231 (54.1%)
Educational level	
Secondary and less	93 (21.8%)
University and higher	339 (78.2%)
Medical student/healthcare professional	
Yes	75 (17.6%)
No	352 (82.4%)
History of depression diagnosis/treatment	
Yes	58 (13.6%)
No	369 (86.4%)
Relative's treatment with antidepressants	
Yes	144 (33.7%)
No	283 (66.3%)
Read or learned about antidepressants	
Yes	195 (45.7%)
No	232 (54.3%)

Table 2 summarizes responses to the 12 knowledge statements on antidepressant use. Misconceptions were common, particularly regarding spontaneous recovery from depression (73.5%), expected onset of antidepressant effect (65.7%), and addiction (74.5%). Stigma-related beliefs were also frequently endorsed, including that antidepressants change personality (82.2%) and may negatively affect marriage prospects (70.3%). Additional items showed mixed responses, including beliefs about short-term treatment, lifelong use, ineffectiveness, discontinuation symptoms, and perceived social consequences. 

**Table 2:** Frequency distribution of correct knowledge about antidepressants among the studied participants Correct versus incorrect responses to the 12 antidepressant knowledge statements, presented as n (%). Each item was scored as correct = 1 and incorrect = 0 to calculate the total knowledge score used in subsequent analyses.

Knowledge items	Correct answer n(%)	Incorrect answer n(%)
Depression can disappear or be treated on its own	113(26.5%)	314(73.5%)
The effect of the medication will be immediate upon taking it, and if this is not the case, it means the patient should change the medication	147(34.3%)	280(65.7%)
Starting antidepressants means you will continue using them for life.	232(54.3%)	195(45.7%)
Antidepressants are a short-term treatment	165(38.6%)	262(61.4%)
Antidepressants are ineffective	230(53.9%)	197(46.1%)
Antidepressants cause addiction	109(25.5%)	318(74.5%)
If the patient chooses to stop taking antidepressants, they will experience severe withdrawal symptoms (difficulty sleeping, headache, loss of concentration, etc.).	170(39.8%)	257(60.2%)
Antidepressants have long-term side effects	268(62.8%)	159(37.2%)
Taking antidepressants is a sign of weakness	290(67.9%)	137(32.1%)
Antidepressants will change the patient's personality	76(17.8%)	351(82.2%)
The use of antidepressants can negatively affect reputation and employment	297(69.6%)	130(30.4%)
The use of antidepressants can negatively affect the patient's chances of marriage	127(29.7%)	300(70.3%)

**Figure 2:**
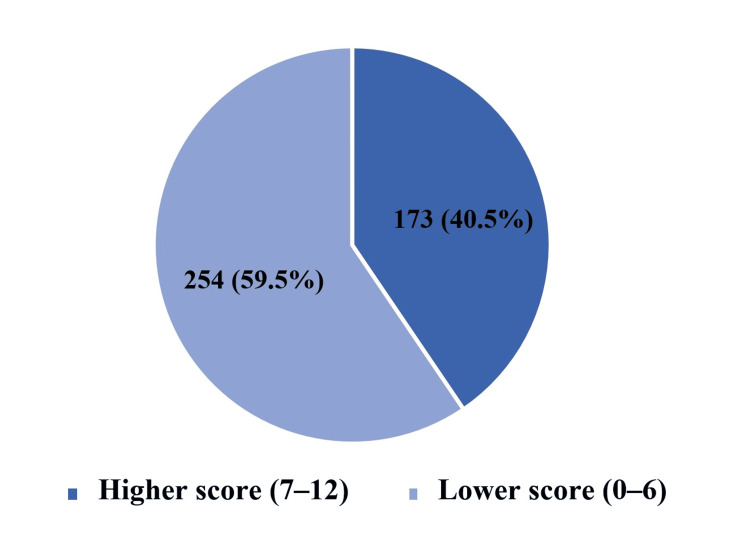
Distribution of knowledge level about antidepressants among the studied participants (N = 427) The figure presents the proportion of Participants were grouped into a higher score (7–12) versus lower score (0–6) based on the pre-specified item coding described in the Methods and Appendix. This figure is descriptive; inferential comparisons by participant characteristics are reported in Tables 3 and 4. This visual summary allows rapid understanding of overall awareness levels in the participants.

Figure 2 shows the overall distribution of knowledge level (good vs poor) in the sample. In Table 3, good knowledge (n = 173) was significantly more common among females than males (p = 0.02), among participants with university education or higher compared with secondary or less (p = 0.04), and among those who had read/learned about antidepressants compared with those who had not (p < 0.0001). No statistically significant differences in knowledge level were observed by age group, medical/healthcare background, history of depression diagnosis/treatment, or having a relative treated with antidepressants (all p > 0.05).

**Table 3:** Comparison of knowledge level by the personal characteristics of the studied participants Knowledge level (good: 7–12 vs. poor: 0–6) by participant characteristics, presented as n (%). Chi-square tests were used for comparisons. *p < 0.05.

Characteristics	Good knowledge N = 173 n(%)	Poor knowledge N = 254 n(%)	Test statistic	P-value
Age in years				
18-25	62 (45.6)	74 (54.4)	χ² = 2.46	0.48
26-35	17 (39.5)	26 (60.5)		
36-45	28 (35.4)	51 (64.6)		
45+	66 (39.1)	103 (60.9)		
Sex				
Male	68 (34.7)	128 (65.3)	χ² = 5.09	0.02*
Female	105 (45.5)	126 (54.5)		
Educational level				
Secondary and less	29 (31.2)	64 (68.8)	χ² = 4.30	0.04*
University and higher	144 (43.1)	190 (56.9)		
Medical student/healthcare professional				
Yes	36 (48.0)	39 (52.0)	χ² = 2.12	0.15
No	137 (38.9)	215 (61.1)		
History of depression diagnosis/treatment				
Yes	25 (43.1)	33 (56.9)	χ² = 0.19	0.66
No	148 (40.1)	221 (59.9)		
Relative's treatment with antidepressants				
Yes	64 (44.4)	80 (55.6)	χ² = 1.39	0.24
No	109 (38.5)	174 (61.5)		
Read or learned about antidepressants				
Yes	104 (53.3)	91 (46.7)	χ² = 24.47	<0.0001*
No	69 (29.7)	163 (70.3)		

Table 4 compares mean knowledge scores across participant characteristics. Mean scores were significantly higher among females than males (6.1 vs 5.1; p < 0.0001), among those with university education or higher (p = 0.002), among participants reporting a relative treated with antidepressants (p = 0.04), and among those who had read/learned about antidepressants (6.4 vs 5.1; p < 0.0001). Mean scores did not differ significantly by age group (p = 0.10), medical/healthcare background (p = 0.07), or history of depression diagnosis/treatment (p = 0.98).

**Table 4:** Comparison of mean knowledge scoring among the studied participants by their characteristics Mean knowledge score by participant characteristics, presented as mean ± SD. Independent t-tests were used for two-group comparisons and one-way ANOVA for age groups. *p < 0.05.

Characteristics	Knowledge scoring Mean ± SD	Test statistic (df)	P value
Age in years			
18-25	6.1 ± 2.1	F(3,423)=2.29	0.10
26-35	5.4 ± 3.1		
36-45	5.4 ± 2.1		
45+	5.5 ± 2.5		
Sex			
Male	5.1 ± 2.5	t(425)= 4.40	<0.0001*
Female	6.1 ± 2.2		
Educational level			
Secondary and less	5.0 ± 2.4	t(425)= 3.20	0.002*
University and higher	5.9 ± 2.4		
Medical student/healthcare professional			
Yes	6.1 ± 2.6	t(425)= 1.67	0.07
No	5.6 ± 2.3		
History of depression diagnosis/treatment			
Yes	5.7 ± 2.6	t(425)= 0.00	0.98
No	5.7 ± 2.4		
Relative's treatment with antidepressants			
Yes	6.0 ± 2.4	t(425)= 2.04	0.04*
No	5.5 ± 2.4		
Read or learned about antidepressants			
Yes	6.4 ± 2.3	t(425)= 5.68	<0.0001*
No	5.1 ± 2.4		

## Discussion

This study assessed public awareness and attitudes regarding antidepressant use in Al-Madinah. Overall, the findings indicate substantial variation in understanding across key aspects of depression and antidepressant treatment. Notably, 73.5% of participants believed depression can resolve on its own, and 65.7% believed antidepressants take immediate effect, reflecting gaps in expectations about depression management and the typical onset of antidepressant benefit. Compared with a study conducted in Riyadh, 21.4% believed depression can resolve independently, and 38.2% were unsure [9], while a study in Hail reported that 65.6% of participants had good general knowledge regarding depression overall [14]. Taken together, these differences likely reflect variation in study focus (general depression vs. antidepressant-specific beliefs), instruments/scoring, and sampling approaches across settings.

Age-related differences were not statistically significant in our sample, although participants aged 18-25 years showed the highest knowledge level (45.6% classified as aware). Similarly, the Riyadh study reported no significant age-group differences [9], whereas the Hail study found significant age differences in depression awareness (68.1% good awareness in ages 18-24 vs. 53.8% in >55) [14]. A study in Bangladesh also reported a negative association between age and awareness, with each 10-year increase associated with 17% lower prevalence of awareness [11]. Overall, the mixed age findings across studies may reflect differences in the constructs measured (antidepressant beliefs vs. general depression awareness) and age categorization rather than a true inconsistency.

Sex differences were observed in our study, with females showing better knowledge than males (p = 0.02); 45.5% of females (105 participants) were classified as aware compared with 34.7% of males (68 participants). In contrast, the Riyadh study found no statistically significant sex difference (p = 0.067), although males had a higher proportion with adequate knowledge (57.7% vs. 48.6%) [9]. Other regional evidence is mixed: a Qatar study reported better knowledge, beliefs, and attitudes toward mental illness among men than women [15], whereas the Hail study found higher awareness among females (68.9%) than males (62.8%) with a significant difference (p = 0.027) [14]. These variations likely reflect both sociocultural influences (e.g., gendered norms in discussing mental health and help-seeking) and methodological differences in sampling and measurement across studies.

Higher education was associated with better antidepressant knowledge in our study (p = 0.04), consistent with prior work. In the Riyadh study, education was the only factor significantly associated with knowledge of antidepressants (p = 0.039) [9]. Related findings have been reported among students in Jordan, where later years of medical training and higher GPA were associated with greater knowledge and more favorable attitudes toward antidepressants [16]. Similarly, the Bangladesh study reported substantially higher awareness among those with at least secondary education compared with those with primary or no education [11]. Overall, the pattern across studies supports education as an important correlate of awareness and understanding of mental health treatments.

Misconceptions about dependence were prominent in our sample: 74.5% believed antidepressants cause addiction. This aligns with a Dammam study in which 71% believed antidepressants cause addiction, while many stated that this belief would not prevent medication use [17]. In contrast, a Hail study reported a lower proportion (45.98%) endorsing this belief [14], and a Qatar study found differences by gender in beliefs about psychiatric medication causing addiction (54.7% vs. 63.5%; p < 0.001) [15]. Although antidepressants are not addictive in the traditional sense, abrupt discontinuation can cause withdrawal symptoms; therefore, misunderstanding in this area may affect help-seeking and adherence. Consistent with this, stigma-linked beliefs were also common in our study: 70.3% believed antidepressants negatively impact marriage prospects, and 30.4% believed they negatively affect reputation and employment.

Stigma-related perceptions appeared to be a persistent theme across settings. A cross-cultural study across 16 Arab countries identified stigma as a significant barrier contributing to negative attitudes toward help-seeking for mental illness [18]. In Dammam, 255 (65.6%) participants believed that patients on antidepressants are concerned about social stigma (with higher agreement among females), whereas a smaller subgroup believed stigma is not associated with antidepressant use, showing a significant gender difference [17]. A Qatar study similarly reported that females were more likely to feel ashamed to disclose having a family member with mental illness compared with males [15]. Meanwhile, Hail data suggested relatively more accepting attitudes toward depression (63.9% willing to work with individuals with depression and 62.7% willing to form friendships) [14], and Riyadh findings showed that 44.3% disagreed that antidepressants negatively affect reputation and career (25.9% agreed) [9]. Collectively, these results suggest stigma varies across contexts but remains relevant to public perceptions and potential treatment-related decisions.

Personality-related stigma was especially notable in our findings: 82.2% believed antidepressants alter personality, and 32.1% believed taking antidepressants is a sign of weakness. In Riyadh, 22.2% believed individuals who take antidepressants have a weak personality [9], and in Hail, 27.9% believed individuals with depression have weak personalities [14]. Overall, these patterns indicate that stigmatizing beliefs about antidepressant use and mental illness remain prevalent and may require targeted correction through culturally appropriate messaging and communication strategies.

Limitations of the study

This study has several limitations. First, participants were recruited using convenience (non-probability) sampling in Al-Madinah, which may introduce selection bias and limit generalizability; therefore, the sample should not be considered statistically representative of the wider population. Second, the cross-sectional design prevents causal inference and only allows assessment of associations at one time point. Third, although the questionnaire was adapted from a published instrument and piloted for clarity, full psychometric validation (e.g., reliability/validity testing) was not performed. Fourth, the study assessed knowledge and attitudes only and did not measure treatment-seeking behavior or health outcomes, so no conclusions can be drawn about downstream effects. Finally, results are self-reported; multiple comparisons may increase the risk of false-positive findings, and some subgroup analyses may have been underpowered, so non-significant results should not be interpreted as evidence of no association.

Recommendations and implications of the study

The results suggest notable misconceptions and knowledge gaps about antidepressants and their mechanisms of action among surveyed participants in the Al-Madinah Region. Many believed depression can resolve without treatment and expected antidepressants to work immediately, highlighting a limited understanding of depression management and the time to benefit, and supporting targeted public education and clinician-patient communication about appropriate antidepressant use.

The following recommendations are proposed: Encourage scientific research to study the factors influencing public perceptions of mental disorders, which will help in developing effective strategies to increase awareness. Hold parent meetings in schools during World Mental Health Day or World Depression Day to raise awareness about depression and its treatment through expert lectures, discussion sessions, distribution of educational brochures, and enhanced communication between families and schools to support students’ mental health. Engage digital media platforms and social influencers in spreading awareness by hosting psychiatrists in live broadcasts, educational interviews, or social media discussions to ensure the dissemination of accurate information to a broad audience.

Implications of the Study: This study provides a descriptive baseline of community knowledge and attitudes that can help relevant authorities, such as the Ministry of Health and local health educators, to prioritize content areas and tailor messaging to the misconceptions identified in the surveyed population. Future research is needed to examine whether addressing these misconceptions is associated with changes in stigma, help-seeking, or other outcomes.

## Conclusions

In this cross-sectional online survey of adults in Al-Madinah (N = 427), misconceptions about antidepressant use were common, including beliefs that depression can resolve on its own (73.5%), antidepressants work immediately (65.7%), and antidepressants cause addiction (74.5%). Stigma-related beliefs were also frequent, such as the perception that antidepressants change personality (82.2%) or may negatively affect marriage prospects (70.3%) and employment opportunities (30.4%). Awareness scores were higher among females, participants with university education, and those who had previously read or learned about antidepressants (p < 0.05). Because the sample was recruited through non-probability convenience sampling, findings should be interpreted as descriptive of the surveyed respondents rather than the wider population; however, they highlight specific misconceptions and stigma themes that may inform targeted local education and clinician-patient communication and should be confirmed in future studies using more representative sampling.

## Appendices

Questionnaire and scoring key

Response options for belief statements: Agree / Maybe / Disagree / I do not know.

Scoring rule: For each statement, one response option is coded as evidence-consistent / non-stigmatizing (correct)(score = 1). All other options are coded (incorrect) (score = 0) (including Maybe and I do not know). The total awareness score is the sum across the 12 items (range: 0-12).

A1. Scoring key for the 12 statements (non-table format)

Statement: Depression can disappear or be treated on its own Score = 1 response: Disagree Rationale: Depression may remit in some mild cases, but this statement reflects the misconception that professional assessment/support is generally unnecessary.

Statement: The effect of antidepressant medication will be immediate; if not, the patient should change the medication Score = 1 response: Disagree Rationale: Full therapeutic benefit typically takes weeks; “immediate effect” (and immediate switching) reflects a misconception.

Statement: Starting antidepressants means lifelong use Score = 1 response: Disagree Rationale: Duration of treatment varies; lifelong use is not inevitable.

Statement: Antidepressants are a short-term treatment Score = 1 response: Disagree Rationale: Course length varies by severity/recurrence; it is not simply a short-term fix for all, and often continues beyond symptom improvement.

Statement: Antidepressants are ineffective Score = 1 response: Disagree Rationale: Response varies, but antidepressants are effective for many; a blanket “ineffective” statement is inaccurate.

Statement: Antidepressants cause addiction Score = 1 response: Disagree Rationale: Antidepressants are not addictive in the classic sense; this item targets confusion between addiction and discontinuation symptoms.

Statement: Stopping antidepressants causes severe withdrawal symptoms Score = 1 response: Disagree Rationale: Discontinuation symptoms vary; the statement implies severe symptoms are inevitable for all.

Statement: Antidepressants have long-term side effects Score = 1 response: Agree Rationale: Long-term use may be associated with persistent or late-emerging adverse effects; this item reflects general safety awareness (possibility, not inevitability).

Statement: Taking antidepressants is a sign of weakness Score = 1 response: Disagree Rationale: Stigma-related belief; the evidence-consistent stance is non-stigmatizing.

Statement: Antidepressants will change the patient’s personality Score = 1 response: Disagree Rationale: Medications can affect symptoms/mood; the statement implies inevitable or dramatic personality change.

Statement: Using antidepressants can negatively affect reputation/employment Score = 1 response: Disagree Rationale: Stigma/social consequence belief; reflects perceptions rather than direct medication effects.

Statement: Using antidepressants can negatively affect chances of marriage Score = 1 response: Disagree Rationale: Stigma/social consequence belief; reflects perceptions rather than direct medication effects.

A2. Questionnaire items (clean transcript)

Section 1. Demographics

Sex: Male / Female

Age group: 18-25 / 26-35 / 36-45 / ≥45

Education level: Secondary or less / University or higher

Section 2. Background and exposure

Are you a medical student or healthcare professional? (Yes/No)

Have you ever been diagnosed with or treated for depression? (Yes/No)

Do you have a close relative treated with antidepressants? (Yes/No)

Have you ever read or learned about antidepressants? (Yes/No)

If yes, specify the source (select all that apply): TV/Newspaper; Medical books; Internet; Social media; Other

Section 3. Belief statements (Agree / Maybe / Disagree / I do not know)

Depression can disappear or be treated on its own.

The effect of antidepressant medication will be immediate; if not, the patient should change the medication.

Starting antidepressants means lifelong use.

Antidepressants are a short-term treatment.

Antidepressants are ineffective.

Antidepressants cause addiction.

Stopping antidepressants causes severe withdrawal symptoms.

Antidepressants have long-term side effects.

Taking antidepressants is a sign of weakness.

Antidepressants will change the patient’s personality.

Using antidepressants can negatively affect reputation/employment.

Using antidepressants can negatively affect the chances of marriage.
